# The structure of NMB1585, a MarR-family regulator from *Neisseria meningitidis*

**DOI:** 10.1107/S174430910900414X

**Published:** 2009-02-26

**Authors:** Charles E. Nichols, Sarah Sainsbury, Jingshan Ren, Thomas S. Walter, Anil Verma, David K. Stammers, Nigel J. Saunders, Raymond J. Owens

**Affiliations:** a The Division of Structural Biology, Wellcome Trust Centre for Human Genetics, University of Oxford, Roosevelt Drive, Oxford OX3 7BN, England; b Oxford Protein Production Facility, Wellcome Trust Centre for Human Genetics, University of Oxford, Roosevelt Drive, Oxford OX3 7BN, England; c The Bacterial Pathogenesis and Functional Genomics Group, The Sir William Dunn School of Pathology, University of Oxford, South Parks Road, Oxford OX1 3RE, England

**Keywords:** MarR, *Neisseria meningitidis*, transcription factors

## Abstract

The structure of the MarR-family transcription factor NMB1585 from *Neisseria meningitidis* has been solved using data extending to a resolution of 2.1 Å. Overall, the dimeric structure resembles those of other MarR proteins, with each subunit comprising a winged helix–turn–helix (wHtH) domain connected to an α-helical dimerization domain. The spacing of the recognition helices of the wHtH domain indicates that NMB1585 is pre-configured for DNA binding, with a putative inducer pocket that is largely occluded by the side chains of two aromatic residues (Tyr29 and Trp53). NMB1585 was shown to bind to its own promoter region in a gel-shift assay, indicating that the protein acts as an auto-repressor.

## Abstract 2

The structure of the MarR-family regulator NMB1585 from *N. meningitidis* has been solved using data extending to 2.1 Å resolution.

## 1. Introduction

In *Escherichia coli*, the multiple antibiotic resistance (*mar*) locus regulates the expression of proteins that confer resistance to numerous exogenous factors such as antibiotics, organic solvents, oxidative stress and disinfectants (Alekshun *et al.*, 2001 ▶; Ellison & Miller, 2006 ▶; Sulavik *et al.*, 1995 ▶). Resistance to these antimicrobial agents or environments is believed to be determined primarily through the control of efflux pumps with a range of specificities, the expression of which is controlled locally by the binding of MarR to its cognate DNA, preventing initiation of gene transcription and thereby acting as a repressor (Alekshun *et al.*, 2001 ▶).

*E. coli* MarR was the first of the MarR-family regulators to be described and forms the archetype for a family of homologous transcriptional regulators which are widely distributed amongst both archaea and prokaryotes. Some of these homologues are also known to control *mar*-type efflux pump operons, *e.g.* FarR in *Neisseria gonorrhoeae*, which controls the expression of the FarAB efflux pump mediating resistance to long-chain fatty acids (Lee *et al.*, 2003 ▶), and MgrA in *Staphylococcus aureus*, which controls the expression of NorA, a multidrug transporter responsible for resistance to fluoro­quinolones (Truong-Bolduc *et al.*, 2005 ▶). In other cases, homologues have been recruited to different systems and regulate tissue-specific activities such as the adhesive properties of cells, haemolytic properties and regulation of protease expression (Ludwig *et al.*, 1995 ▶; Marklund *et al.*, 1992 ▶; Perego & Hoch, 1988 ▶; Saridakis *et al.*, 2008 ▶).

Knowledge of the three-dimensional structures of the MarR-family regulators has contributed to understanding their mechanism of action. To date, the structures of more than 20 MarR-family regulators have been solved and deposited in the Protein Data Bank, including those of *E. coli* MarR (Alekshun *et al.*, 2001 ▶), *Bacillus subtilis* OhrR in the unliganded state and bound to its cognate DNA (Hong *et al.*, 2005 ▶), *Deinococcus radiodurans* HucR (Bordelon *et al.*, 2006 ▶), *Enterococcus faecalis* SlyA (Wu *et al.*, 2003 ▶), *Methanobacterium thermoautotrophicum* MarR in the unliganded state and with salicylate bound (Saridakis *et al.*, 2008 ▶), *Pseudomonas aeruginosa* MexR (Lim *et al.*, 2002 ▶), *Sulfolobus tokodaii* EmrR (Miyazono *et al.*, 2007 ▶) and *Xanthomonas campestris* MarR (Chin *et al.*, 2006 ▶). Many of these homologues share less than 20% sequence identity, but they all possess the same core fold. The proteins are homodimers comprising a largely helical dimerization domain linked to a DNA-binding domain that contains a winged helix–turn–helix motif. MarR proteins repress the activity of their target genes by binding as dimers to pseudopalindromic sequences in the −10 region of the regulated promoters. The repressor activity of MarR proteins is modulated by co-inducer binding, or in the case of OhrR oxidation of cysteines disrupting disulfide-bridge formation, both of which lead to a major rearrangement in the dimerization region such that the spacing of the DNA-binding domains is significantly altered, preventing DNA recognition.

*Neisseria meningitidis* encodes two MarR-family repressors, NMB1853, a homologue of FarR found in *N. gonorrhoeae* which presumably also controls expression of the FarRAB efflux pump, and a second MarR, NMB1585, of unknown function. In *N. gonorrhoeae* the close homologue of NMB1585 encoded by NGO1244 has been shown to be part of the RpoH regulon and is upregulated in response to temperature stress (Gunesekere *et al.*, 2006 ▶). Given the potential importance of NMB1585 in the pathophysiology of *N. meningitidis*, we have targeted this protein for structural studies and in this report we describe the structure at 2.1 Å resolution.

## 2. Materials and methods

### 2.1. Protein production and crystallization

The NMB1585 expression construct was generated by means of ligation-independent cloning using Gateway technology (Invitrogen). NMB1585 was amplified from genomic DNA (*N. meningitidis* strain MC58) with KOD HiFi polymerase (Novagen) using the forward primer 5′-GGGGACAAGTTTGTACAAAAAAGCAGGCTTCCT­GGAAGTTCTGTTCCAGGGCCCGATGAACCAACTCGACCA­ACTTGGC-3′ and the reverse primer 5′-­GGGGACCACTTTGTACAAGAAAGCTGGGTCTCACTATTTTTTATTTTCCGAGATT­GTTTTTTC-3′. The PCR product was purified using QIAquick 96 plates (Qiagen) and cloned into the expression vector pDEST17 in two steps according to the manufacturer’s protocol (Invitrogen), resulting in a construct with an N-terminal His tag and 3C protease cleavage site. BP and LR reactions were carried out according to the manufacturer’s instructions. Recombinant LR clones were identified by PCR using a gene-specific forward primer and a T7 reverse primer and verified by DNA sequencing. Protein was produced in *E. coli* strain B834 (DE3). The cells were grown at 310 K in GS96 media (QBiogene) to an *A*_600_ of 0.6, induced by the addition of 0.5 m*M* IPTG and then incubated for a further 20 h at 293 K. The cells were harvested by centrifugation at 6000*g* for 15 min and lysed using a Basic Z Cell Disruptor (Constant Systems Ltd) at 207 MPa in 50 m*M* Tris pH 7.5, 500 m*M* NaCl, 0.2%(*v*/*v*) Tween-20. The protein was purified by nickel-affinity chromatography followed by size-exclusion chromatography using the standard His Affinity-Gel filtration program on the ÄKTA 3D (GE Healthcare). After centrifugation at 30 000*g* for 30 min, the lysate was loaded onto a 1 ml pre-charged HiTrap Chelating Sepharose FF column (GE Healthcare). The column was washed with 50 m*M* Tris pH 7.5, 500 m*M* NaCl, 20 m*M* imidazole. The protein was then eluted in 50 m*M* Tris pH 7.5, 500 m*M* NaCl, 500 m*M* imidazole and injected onto a 16/60 HiLoad Superdex 200 column (GE Healthcare) equilibrated in 20 m*M* Tris pH 7.5, 200 m*M* NaCl. Protein-containing fractions were analyzed on SDS–PAGE gels (Biorad). The N-terminal tag was removed by overnight incubation at 277 K with His-tagged 3C protease (prepared from pET-24/His-3C kindly provided by A. Geerlof, EMBL, Heidelberg). The 3C protease and any uncleaved protein were removed by nickel-affinity chromatography and the protein was concentrated to 9.7 mg ml^−1^ using a Vivaspin 15 concentrator with 5 kDa molecular-weight cutoff (Vivascience) in 20 m*M* Tris pH 7.5, 200 m*M* NaCl, 1 m*M* tris(2-carboxyethyl)phosphine (TCEP). The protein was crystallized using a nanodrop crystallization procedure (Walter *et al.*, 2005 ▶). Crystals were initially obtained in 0.1 *M* HEPES buffer pH 7.5 containing 25%(*w*/*v*) PEG 3350, 0.2 *M* ammonium chloride and growth was optimized by varying the pH of the precipitant by addition of acid/base as described by Walter *et al.* (2005 ▶). The crystals of NMB1585 used for data collection were partially dehydrated/cryoprotected by a three-stage transfer to 40%(*w*/*v*) polyethylene glycol 3350, 15%(*v*/*v*) ethylene glycol and were flash-frozen in a 100 K nitrogen cold stream prior to data collection. Owing to their superior diffraction properties compared with methionine-containing native crystals, data from selenomethionine-labelled crystals were used for both structure determination and refinement.

### 2.2. Crystallography methods

Multiwavelength X-ray diffraction data were collected from selenomethionine-labelled NMB1585 crystals on beamline BM14 at the ESRF, Grenoble. Data were indexed, integrated and scaled using *DENZO* and *SCALEPACK* (Otwinowski & Minor, 1997 ▶; Table 1 ▶). The protein was crystallized in space group *P*2_1_, with two subunits per asymmetric unit. The selenomethionine substructure of the NMB1585 crystal was solved by multiwavelength anomalous dispersion methods using *SHELXD* (Sheldrick, 2008 ▶). Three of four possible selenium sites were thus located and were used to obtain an initial phase set (*SHELXE*; phase extension to 2.1 Å, contrast 0.499, connectivity 0.930, pseudo-free CC 70.64%, mean FOM 0.62). Further density modification and initial model building were then performed with *RESOLVE* (Terwilliger, 2004 ▶), yielding a dimeric starting model with 75% of the expected number of residues and 50% of the sequence threaded. This model was then refined with *CNS* (Brünger *et al.*, 1998 ▶), iterated with several rounds of rebuilding in *O* (Jones *et al.*, 1991 ▶). Final statistics are given in Table 1 ▶. 

### 2.3. Electrophoretic mobility shift assay

A 378 bp probe corresponding to the intergenic sequence between *NMB1584* and *NMB1585* was amplified from genomic DNA using the following pair of PCR primers: forward primer 5′-CGAACAGG­ACGTTTCCGGCG-3′ and reverse primer 5′-CATTGCAAATCA­GGTTGATACGG-3′. A fluorescence-detection method was used for the electrophoretic mobility shift assay (EMSA), as described by the manufacturer (Electrophoretic Mobility Shift Assay Kit, Invitrogen). Briefly, DNA (20 n*M*) was incubated at room temperature with increasing amounts of purified NMB1585 protein (up to 640 n*M* for the dimeric form) in a total volume of 10 µl containing 1× EMSA binding buffer (10 m*M* Tris pH 7.4, 0.1 m*M* dithiothreitol, 0.1 m*M* EDTA, 150 m*M* KCl). Following the addition of 2 µl 6× EMSA gel-loading buffer, the samples were loaded onto a pre-cast tris–borate–EDTA (89 m*M* Tris base, 89 m*M* boric acid, 1 m*M* EDTA pH 8.0) gel (10%; Invitrogen) that had been pre-equilibrated for 15 min at 120 V in ice-cold 0.5× TBE running buffer (Invitrogen). The gels were run at 120 V for 15 min followed by 160 V for a further 70 min. The gels were stained for 20 min in the dark with SYBR Green EMSA nucleic acid gel stain in 1× TBE buffer. After washing twice in distilled water for 10 s each time, the gels were visualized on a UV-light trans­illuminator using the Gene Genius Bio imaging system (Syngene).

## 3. Results and discussion

### 3.1. Overall structure

**Figure 1:**
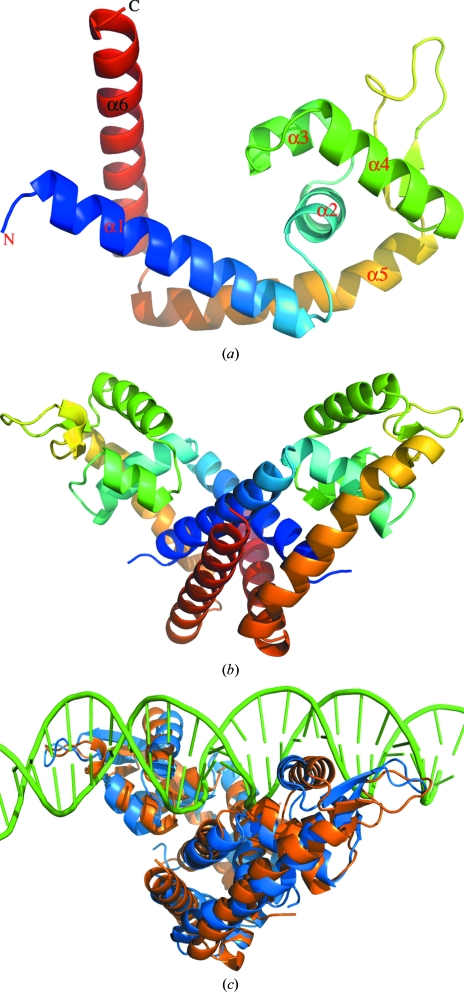
The structure of NMB1585. (*a*, *b*) Ribbon diagrams showing the overall structures of the NMB1585 monomer and dimer; (*c*) superposition of NMB1585 dimer (orange) with the OhrR–DNA complex (PDB code 1z91; blue and green).

The structure of NMB1585 was solved to a resolution of 2.1 Å by the multiple-wavelength anomalous dispersion method using seleno­methionine-substituted protein. Like other members of this family of regulators, the meningococcal MarR monomer structure is pre­dominantly α-helical, with an elongated ‘arm’ domain (α1, α5 and α6) linked to a more compact ‘wing’ domain that contains a winged helix–turn–helix motif (wHtH; topology α2-t-α3-t-α4-β1-W1-β2; Fig. 1 ▶). Consistent with other MarR-family regulators, NMB1585 is dimeric, with the two wHtH motif-containing ‘wing’ domains distal to the dimer interface (Figs. 1 ▶ *a* and 1 ▶ *b*). The dimer interface is thus formed by a symmetrical interaction of the arm domains of the two monomers and involves contacts between both N-terminal and C-­terminal regions (approximately residues 1–25 and 110–142). The orientation of the two monomers in the dimer and their ability to pivot relative to one another has previously been shown to be an essential factor affecting the DNA-binding mode of MarR-type repressors such as MexR (Lim *et al.*, 2002 ▶), HucR (Bordelon *et al.*, 2006 ▶; Wilkinson & Grove, 2005 ▶) and MobR (Hiromoto *et al.*, 2006 ▶). More recently, a comparison of unliganded and DNA-bound forms of OhrR identified clear conformational changes accompanying DNA binding (Hong *et al.*, 2005 ▶), whilst analysis of *M. thermoautotrophicum* MarR (MTH313) crystal structures revealed a large reorientation of the ‘arm’ and ‘wing’ domains accompanying salicylate binding (Saridakis *et al.*, 2008 ▶).

### 3.2. Comparison with other MarR structures

**Figure 2:**
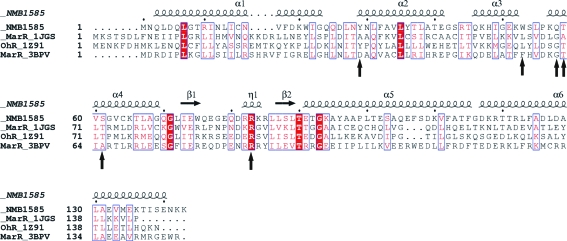
Structure-based alignment of NMB1585 with MarR sequences of known structure. The sequences of NMB1585 (*N. meningitidis*), MarR (*E. coli*; PDB code 1jgs), OhrR (*B. subtilis*; PDB code 1z91) and MTH313 (*M. thermoautotrophicum*; PDB code 3bpv) were aligned using *ClustalW* and displayed with secondary structures using *ESPript*2.2. The residues proposed to be involved in DNA binding (Gln58, Thr59, Ser61, Arg83) and ligand binding (Tyr29, Trp53) are indicated by arrows.

The structure of NMB1585 was superimposed onto that of the *B. subitilis* OhrR–DNA complex and shown to match it closely, with an r.m.s.d. of 2.4 Å for 213 equivalent C^α^ atoms of 274 (Fig. 1 ▶ *c*). Thus, NMB1585 appears to be pre-configured for DNA binding, similar to the structures reported for the HucR regulator of *D. radiodurans* (Bordelon *et al.*, 2006 ▶) and a MarR-family protein from *S. tokodaii* (Kumarevel *et al.*, 2008 ▶). However, this does not appear to be typical amongst other MarR structures (Hong *et al.*, 2005 ▶). By reference to the OhrR structure, the recognition site of NMB1585 is likely to be approximately 20 bp, with each monomer binding into consecutive major grooves of the DNA double helix. The residues in the recognition helix (α4) that are most likely to be involved in binding in the major groove of DNA are Gln58, Thr59 and Ser61 (Fig. 2 ▶). In the OhrR complex, a highly conserved arginine (Arg94) residue makes the key contact between the wing of the wHtH motif and the minor groove of the DNA target and it has been proposed that this represents a generic interaction common to all MarR-family proteins (Hong *et al.*, 2005 ▶; Kumarevel *et al.*, 2008 ▶). The conformation of the wing loop of NMB1585 closely resembles that of OhrR and Arg83 would make a similar minor-groove contact (Figs. 1 ▶ *c* and 2 ▶). It follows that discrimination between different DNA-binding sites must largely depend on the sequence and orientation of the recognition helix (α4) of the HtH motif which contacts the major groove of the DNA.

**Figure 3:**
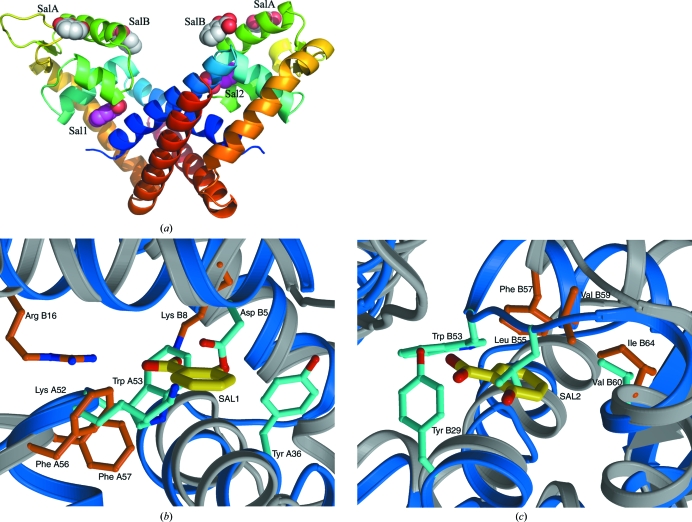
Comparing the salicylate-binding sites of *E. coli* and *M. thermoautotrophicum* MarRs with those of NMB1585. (*a*) Ribbon diagram showing the NMB1585 dimer with the relative salicylate-binding sites in *E. coli* (PDB code 1jgs; SalA and SalB) and *M. thermoautotrophicum* (PDB code 3bpv; Sal1 and Sal2) marked by salicylate molecules drawn as space-filling objects; (*b*) and (*c*) comparison of the *M. thermoautotrophicum* MarR salicylate-binding sites with the corresponding regions of NMB1585 by overlapping the two chains separately; the backbones are shown as ribbons and the side chains as sticks, with *M. thermoautotrophicum* MarR coloured grey and orange and NMB1585 in blue and cyan; the salicylate molecules are shown as thicker sticks and coloured yellow.

A feature of the MarR family is their capacity to bind to a variety of effector molecules, generally phenolic compounds such as sali­cylate; in most cases, this results in a reduction of DNA binding (Wilkinson & Grove, 2006 ▶). The results of cocrystallization experiments have shown that salicylate can bind at two different locations in MarR proteins. In the structure of *E. coli* MarR, a salicylate molecule was observed bound in two surface pockets (termed SalA and SalB) on each subunit that were located either side of the DNA-recognition helix (Alekshun *et al.* (2001 ▶). These positions are indicated in the structure of NMB1585, clearly showing how occupancy is likely to directly interfere with DNA binding (Fig. 3 ▶). However, the residues in *E. coli* MarR that form these surface pockets and interact with the salicylate molecules are not conserved in NMB1585, indicating that binding to this part of the protein is highly unlikely. In contrast, the salicylate-binding pocket identified in MTH313, a MarR protein from *M. thermoautotrophicum*, is conserved (Saridakis *et al.*, 2008 ▶). In the MTH313 structure one salicylate molecule was observed bound to each subunit of the dimer at the interface of the DNA-binding domain and the helical dimerization domain. The two salicylates were observed to interact at different sites within the binding pocket (Fig. 3 ▶). The positions of the bound salicylates in MTH313 have been mapped onto the NMB1585 structure and are shown in Fig. 3 ▶(*a*); detailed views of the two binding sites are shown in the superimposition of the two structures (Figs. 3 ▶ *b* and 3 ▶ *c*). Overlaying of NMB1585 and MTH313 confirms the presence of a potential ligand-binding pocket in NMB1585 at a similar location to those in MTH313 and other MarR proteins (Saridakis *et al.*, 2008 ▶). However, it is clear that the side chains of the residues that line the pocket, notably Tyr29, Tyr36 and Trp53, occupy much of the internal volume of the binding pocket, which would prevent a ligand such as salicylate from binding to this conformation of the protein (Figs. 3 ▶ *b* and 3 ▶ *c*). Therefore, in order for a ligand to bind into the binding pocket of NMB1585 a conformational change would have to occur in the protein so that the dimerization domain moved away from the DNA-binding domain. This would increase the separation of the α2-helix with respect to α3-helix in each subunit, thus opening up the hydrophobic pocket.

### 3.3. DNA binding

**Figure 4:**
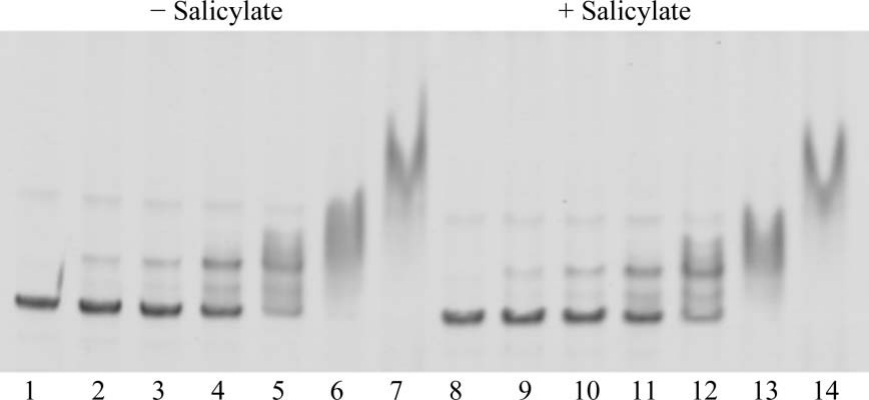
EMSA of NMB1585–DNA complexes. Increasing amounts of purified NMB1585 protein (20–640 n*M*) were incubated with a 378 bp DNA probe (20 n*M*) PCR-amplified from the promoter region of the NMB1585 gene in either the absence (lanes 2–8) or presence (lanes 9–14) of 10 m*M* sodium salicylate. The DNA–protein complexes were analysed by gel electrophoresis on a tris–borate–EDTA poly­acrylamide gel and stained for DNA using SYBR green. Samples are as follows: DNA only (lanes 1 and 8), 20 n*M* protein (lanes 2 and 9), 40 n*M* protein (lanes 3 and 10), 80 n*M* protein (lanes 4 and 11), 160 n*M* protein (lanes 5 and 12), 320 n*M* protein (lanes 6 and 13) and 640 n*M* protein (lanes 7 and 14).

The overall structure of NMB1585 confirms that the protein is a member of the MarR family of transcription repressors. DNA binding was verified experimentally in an EMSA experiment. Purified NMB1585 protein showed concentration-dependent binding to a double-stranded DNA probe corresponding to the region between the end of the upstream gene (*NMB1584*) and the start of the coding sequence for NMB1585 (Fig. 4 ▶). This region contains the promoter for NMB1585 and suggests that, in common with other MarR regulators [*e.g.* FarR (Lee *et al.*, 2003 ▶) and MexR (Evans *et al.*, 2001 ▶)], NMB1585 is an auto-regulator. Two potential DNA–protein com­plexes were observed in the EMSA experiments: a faster migrating species at low protein:DNA ratios and a complex of slower mobility at higher protein:DNA ratios. This suggests that there is more than one binding site for NMB1585 in the region between the *NMB1564* and *NMB1565* genes. Typically, MarR regulators bind to relatively short (pseudo)palindromic sequences consistent with the dimeric structure of the proteins, although the lengths of the inverted repeats and the spacing between half-sites is variable (Wilkinson & Grove, 2006 ▶). Further experiments would be required, for example DNA footprinting, to identify the cognate DNA-binding sites of NMB1585. Interestingly, the addition of salicylate, a prototypical MarR ligand, did not affect the formation of the protein–DNA complexes (Fig. 4 ▶), suggesting that the protein does not interact with salicylate, in contrast to *E. coli* MarR (Alekshun *et al.*, 2001 ▶) and MTH313 (Saridakis *et al.*, 2008 ▶). This may be explained by the occluded nature of the putative binding site observed in the crystal structure of NMB1585 and suggests that the protein may adopt this conformation in solution.

The physiological role of NMB1585 has not been characterized and therefore the identity of any natural ligand(s) that may modulate its activity is unknown. Intriguingly, the gene immediately downstream of NMB1585 is annotated as a potential integral membrane protein (NMB1586) classified as a component of an ABC-type multidrug transport system, ATPase and permease. Transcription analysis of a NMB1585-knockout strain shows an increase in NMB1586 transcript expression in the absence of NMB1585 (N. J. Saunders, unpublished data). Given the role of MarR-family proteins in the regulation of the expression of efflux systems, it is tempting to speculate that NMB1585 may be a repressor of a transport protein involved in the export of xenobiotic compounds from *Neisseria*.

In conclusion, we describe the crystal structure of meningococcal MarR, which represents a highly adaptable fold widely used in transcriptional regulation in many bacteria with particular significance in controlling responses to changes in their chemical environment.

## Supplementary Material

PDB reference: NMB1585, 3g3z, r3g3zsf

## Figures and Tables

**Table 1:** X-ray data-collection and refinement statistics Values in parentheses are for the highest resolution shell.

Data set	Peak	Inflection	Remote
Data-collection details			
X-ray source	ESRF BM14		
Wavelength (Å)	0.97889	0.97912	0.90777
Space group	*P*2_1_		
Unit-cell parameters (Å, °)	*a* = 35.00, *b* = 64.37, *c* = 61.07, β = 91.12		
Resolution range (Å)	30.0–2.10 (2.18–2.10)		
Unique reflections	14550 (835)	14325 (715)	14294 (763)
Completeness† (%)	90.6 (52.4)	88.9 (44.4)	89.4 (48.1)
Redundancy	8.9 (4.6)	3.3 (1.9)	3.2 (1.9)
Average *I*/σ(*I*)	24.9 (2.0)	15.6 (1.3)	17.4 (1.7)
*R*_merge_	0.107 (0.494)	0.072 (0.381)	0.063 (0.264)
Refinement statistics			
Resolution range (Å)			30.0–2.10
No. of reflections (working/test)			13531/734
*R* factor‡ (*R*_work_/*R*_free_)			0.203/0.266
No. of atoms (protein/water)			2254/109
R.m.s. bond-length deviation (Å)			0.008
R.m.s. bond-angle deviation (°)			1.0
Mean *B* factor (protein/water) (Å^2^)			26/27

† The data are essentially complete to 2.3 Å resolution.

‡ *R*_work_ and *R*_free_ are defined by *R* = 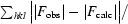 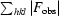, where *hkl* are the indices of the reflections (used in refinement for *R*_work_; 5% not used in refinement for *R*_free_) and *F*_obs_ and *F*_calc_ are the structure factors deduced from measured intensities and calculated from the model, respectively.
